# Editorial: Comorbidity in multiple sclerosis and related disorders

**DOI:** 10.3389/fimmu.2024.1469614

**Published:** 2024-08-19

**Authors:** Mahdi Barzegar, Jelena Drulovic, Viviana Nociti

**Affiliations:** ^1^ Isfahan Neurosciences Research Center, Isfahan University of Medical Sciences, Isfahan, Iran; ^2^ Department of Neurology, School of Medicine, Isfahan University of Medical Sciences, Isfahan, Iran; ^3^ Clinic of Neurology, University Clinical Center of Serbia, Belgrade, Serbia; ^4^ Faculty of Medicine, University of Belgrade, Belgrade, Serbia; ^5^ Fondazione Policlinico Universitario ‘A. Gemelli’ IRCCS, Rome, Italy; ^6^ Centro di Ricerca per la Sclerosi Multipla (CERSM) ‘Prof. Anna Paola Batocchi’, Università Cattolica, Rome, Italy

**Keywords:** multiple sclerosis, comorbidity, neuromyelities optica spectrum disorders, CNS demyelinating diseases, autoimmune diseases

Multiple sclerosis (MS) and related disorders such as neuromyelitis optica spectrum disorders (NMOSD) and myelin oligodendrocyte glycoprotein antibody-associated disease (MOGAD) are debilitating diseases affecting the central nervous system (CNS). These diseases commonly affect young adults, leading to morbidity and mortality, which causes many complications for patients (1).

Comorbidity refers to the co-existence of multiple physical or psychological health conditions in the same person at the same time (2). MS patients are more vulnerable to developing comorbidities than the general population (3). This co-occurrence can be due to direct causation, associated risk factors, or disease heterogeneity. Direct causation refers to the mechanism of a primary disease or its treatment leading to another disease. Common risk factors, such as environmental factors, smoking, and genetics, can also lead to the development of other medical conditions. Some disease-modifying therapies (DMTs) may alter the risk of developing comorbidities (4).

Various comorbidities, such as cancers, autoimmune diseases, sleep disorders, cardiovascular disorders, and psychiatric disorders, occur in persons with MS or related conditions (5, 6). These comorbidities are linked to adverse health outcomes, increased medical costs, and decreased quality of life. Comorbidities are also associated with delays in MS diagnosis, severe outcomes, and increased mortality (7). The current Research Topic, “Comorbidity in Multiple Sclerosis and Related Disorders,” includes eight papers that investigate the patterns of comorbidities in demyelinating disorders, the impact of comorbidities on these diseases, and the effect of disease and comorbidity-related treatments on MS outcomes.

Marrie et al. conducted a literature review study to update the latest evidence regarding the prevalence, etiology, and effects of comorbidity in MS patients, as well as the management of MS in the context of comorbidity. Drulovic et al. performed an international multicentric study to assess the impact of epilepsy/seizures on the quality of life of MS patients, finding a worse quality of life in MS patients with these comorbidities. Holm et al. compared the prevalence of comorbidities between MS patients aged 50 years or older and an age- and sex-matched control group from the general population, finding a greater burden of comorbidity, assessed by Charlson Comorbidity Index, and a lower 5-year survival rate, in MS patients. Brüggemann et al. studied the societal costs of polypharmacy in German MS patients, describing an association between polypharmacy and the socioeconomic burden of MS. Samadzadeh et al. submitted a protocol for an international multicentric study that plans to prospectively assess the effect of comorbidities on MS, NMOSD, and MOGAD.

The impact of comorbidities on other CNS disorders was also evaluated in three additional studies. Balshi et al. found that antinuclear, thyroperoxidase, thyroglobulin, and antiparietal cell autoantibodies were the most common autoantibodies in Stiff Person Syndrome Spectrum Disorders (SPSD). Autoimmune thyroiditis, insulin-dependent diabetes mellitus, and pernicious anemia, were the most common autoimmune comorbidities in these patients. Yang et al. showed that the neutrophil percentage to albumin ratio and high cervical cord lesions were predictors for the occurrence of pulmonary infection in patients with severe myelitis. Ke et al. reported the demographic and clinical characteristics of 34 cases with autoimmune glial fibrillary acidic protein astrocytopathy (GFAP-A).

In conclusion, these eight studies cover a diverse range of patients with MS or related disorders and advance our knowledge of the effect of comorbidity on these diseases. These findings offer a comprehensive view and provide the latest evidence on comorbidity and demyelinating diseases.
